# NMR Structure of Temporin-1 Ta in Lipopolysaccharide Micelles: Mechanistic Insight into Inactivation by Outer Membrane

**DOI:** 10.1371/journal.pone.0072718

**Published:** 2013-09-09

**Authors:** Rathi Saravanan, Mangesh Joshi, Harini Mohanram, Anirban Bhunia, Maria Luisa Mangoni, Surajit Bhattacharjya

**Affiliations:** 1 School of Biological Sciences, Nanyang Technological University, Singapore, Singapore; 2 Istituto Pasteur-Fondazione Cenci Bolognetti, Dipartimento di Scienze Biochimiche Università La Sapienza, Roma, Italy; National Institute for Medical Research, Medical Research Council, London, United Kingdom

## Abstract

**Background:**

Antimicrobial peptides (AMPs) play important roles in the innate defense mechanism. The broad spectrum of activity of AMPs requires an efficient permeabilization of the bacterial outer and inner membranes. The outer leaflet of the outer membrane of Gram negative bacteria is made of a specialized lipid called lipopolysaccharide (LPS). The LPS layer is an efficient permeability barrier against anti-bacterial agents including AMPs. As a mode of protection, LPS can induce self associations of AMPs rendering them inactive. Temporins are a group of short-sized AMPs isolated from frog skin, and many of them are inactive against Gram negative bacteria as a result of their self-association in the LPS-outer membrane.

**Principal Findings:**

Using NMR spectroscopy, we have determined atomic resolution structure and characterized localization of temporin-1Ta or TA (FLPLIGRVLSGIL-amide) in LPS micelles. In LPS micelles, TA adopts helical conformation for residues L4-I12, while residues F1-L3 are found to be in extended conformations. The aromatic sidechain of residue F1 is involved in extensive packing interactions with the sidechains of residues P3, L4 and I5. Interestingly, a number of long-range NOE contacts have been detected between the N-terminal residues F1, P3 with the C-terminal residues S10, I12, L13 of TA in LPS micelles. Saturation transfer difference (STD) NMR studies demonstrate close proximity of residues including F1, L2, P3, R7, S10 and L13 with the LPS micelles. Notably, the LPS bound structure of TA shows differences with the structures of TA determined in DPC and SDS detergent micelles.

**Significance:**

We propose that TA, in LPS lipids, forms helical oligomeric structures employing N- and C-termini residues. Such oligomeric structures may not be translocated across the outer membrane; resulting in the inactivation of the AMP. Importantly, the results of our studies will be useful for the development of antimicrobial agents with a broader spectrum of activity.

## Introduction

Ribosomally-made cationic antimicrobial peptides (AMPs) are the first line of innate defense of almost all living organisms against microbial pathogens [1], [2], [3], [4]. Several AMPs have a potent and quick activity against bacteria, both Gram-negatives and Gram-positives including multidrug resistant strains, viruses, fungi, parasites and cancer cells [5], [6]. Despite differences in their conformation and length, most naturally occurring AMPs are able to select micro-organisms and physically permeate their membrane making it difficult for them to develop resistance. Therefore, AMPs have been considered as promising lead compounds for the generation of a new class of antibiotics [7], [8], which is urgently needed, due to the growing emergence of resistant microbes to the available drugs. [9], [10], [11].

The membrane-AMP interactions are initiated by an ionic bonding between the positively charged residues of AMPs and the anionic head groups of the microbial membrane phospholipids (which differ from those of the electrically neutral mammalian cell membrane).This initial binding event may lead to an insertion of the hydrophobic residues of AMPs into the non-polar core of membranes [9], [10], [11], [12] Remarkably, the amphipathic character of AMPs in complex with phospholipid bilayers is a critical parameter for their membranolytic activity [9], [10], [11], [12]. In order to exert their lethal effect, AMPs need to associate with the cytosolic (inner) or plasma membrane of bacteria. Typically, in Gram-positive bacteria, the plasma membrane is surrounded by a cell wall made of a thick peptidoglycan layer. Differently, in Gram-negatives, the peptidoglycan layer is thinner and protected by an asymmetric lipid bilayer named outer membrane (OM). The inner leaflet of the OM contains phospholipids similar to those of the inner membrane, but the outer leaflet of the OM is predominantly composed of lipopolysaccharide (LPS). Importantly, the LPS layer acts as a permeability barrier against a variety of molecules including antibiotics and AMPs [13], [14], [15], [16]. Consequently, to be strongly active against Gram-negative bacteria AMPs should be able to efficiently destabilize the LPS-OM [17], [18], [19], [20]. The chemical structure of LPS can be distinguished into three separate domains: (i) the relatively conserved lipid A, consisting of five to six fatty acyl chains linked to two phosphorylated glucosamine residues (ii) the core oligosaccharide segment covalently linked to lipid A moiety, and (iii) a highly diverse polysaccharide chain known as the O-antigen [21]. LPS barrier is believed to be stabilized by LPS-associated cations (mainly Mg^++^) through salt bridges neutralizing the repulsive forces of adjacent LPS molecules. Translocation of AMPs across the OM has been proposed to occur by a ‘self-promoted’ uptake mechanism involving displacement of Mg^++^ cations by the cationic AMPs and the formation of ionic interactions with the negatively charged LPS layer [22]. Recent studies have demonstrated that AMPs may assume specific structures in complex with LPS to disrupt the LPS OM barrier [23], [24], [25], [26], [27]. LPS, also termed as endotoxin, is a pivotal agent for sepsis and septic shock syndrome in humans [28], [29]. There are approximately 120,000 sepsis deaths annually in the USA [30], [31]. LPS binds to a pattern recognition receptor (TLR4) on the surface of macrophages and monocytes and activates well orchestrated signaling cascades releasing cytokines e.g. TNF-α, IL-6, IL-1. These cytokines from immune cells are essential to clear bacterial infections. However, over production of cytokines caused by an excess of LPS often leads to a damage of the host tissues and organs [32], [33]. As sepsis and septic shock is a challenging issue in healthcare, studies on the structures and interactions of AMPs with LPS are a fundamental issue to develop new anti-infective agents with the ability to both kill microbes and neutralize the toxic effect of their LPS [15], [19], [34], [35].

Temporins are a group of short AMPs (8 to 14 amino acid long) found in skin secretion of frogs [36], [37], [38]. Their primary structures are rich in non-polar residues with a fewer cationic and polar amino acids, yielding a net charge of 0 to +3 at neutral pH [36], [37]. Temporins are highly active against Gram positive bacteria and fungi. However, several members of this family, except for temporin-1Tl (TL) and temporin-1Dra, show a limited activity against Gram-negative strains [36], [37]. Mangoni and coworkers demonstrated that LPS from *Escherichia coli* O111:B4 induces self-associations of TA (and also temporin-1Tb, TB) [39], [40]. This should inhibit the peptide's translocation across the OM into the target inner membrane, resulting in reduced antimicrobial activity [39], [40]. A synergistic mechanism within two pairs of temporins (TA+TL and TB+TL) in overcoming bacterial resistance owing to the LPS barrier has been recently described. It relates to the ability of TL to prevent the LPS-induced self-association of TA and TB and hence to allow them to overcome the LPS barrier and explicate their toxic effect on Gram negative bacteria, perturbing the plasma membrane [39], [40], [41]. Notably, due to their short size, temporins are attractive scaffold for the development of novel antibiotics and antiendotoxic agents [42], [43], [44], [45], [46], [47]. However, although TL has a broad spectrum of activity, it is toxic towards human erythrocytes at its microbicidal concentrations, thus limiting its potential applications as therapeutic [48]. On the other hand, TA is not hemolytic but with a weak activity against Gram-negative bacteria when used alone [44], [46]. Since OM permeability significantly controls the antibacterial activity of temporins, it is highly desirable to determine both the structure and interactions of these AMPs in LPS lipids [41]. Here, we have investigated atomic resolution structures of TA, in LPS micelles. Importantly, besides shedding light into the molecular mechanism underlying the OM permeabilization, the results of our studies provide a valuable contribution for the design and development of non-toxic temporin analogs with a broader spectrum of antimicrobial activity.

## Results

### NMR studies of TA in free solution and in LPS Micelles

**Figure 1:**
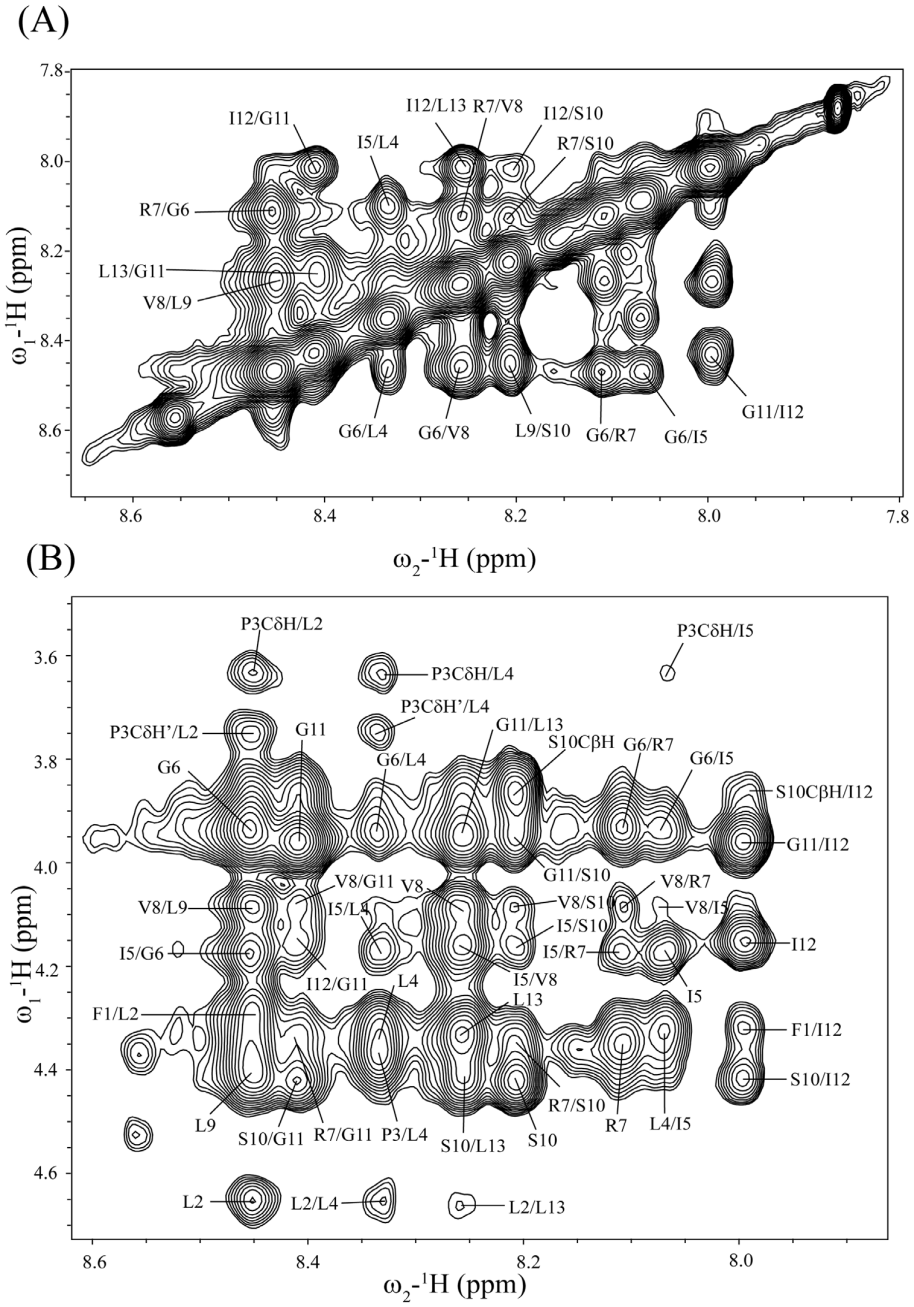
tr-NOESY spectra of TA in LPS micelles. (panel A) A section of the two-dimensional ^1^H-^1^H NOESY spectrum of TA, in aqueous solution containing LPS micelles, showing NOE correlation among backbone amide proton resonances. (panel B) A section of the two-dimensional ^1^H-^1^H NOESY spectrum of TA, in aqueous solution containing LPS micelles, showing NOE correlation among backbone amide proton resonances (along ω2 dimension) with CαH proton resonances (along ω1 dimension).

**Figure 2:**
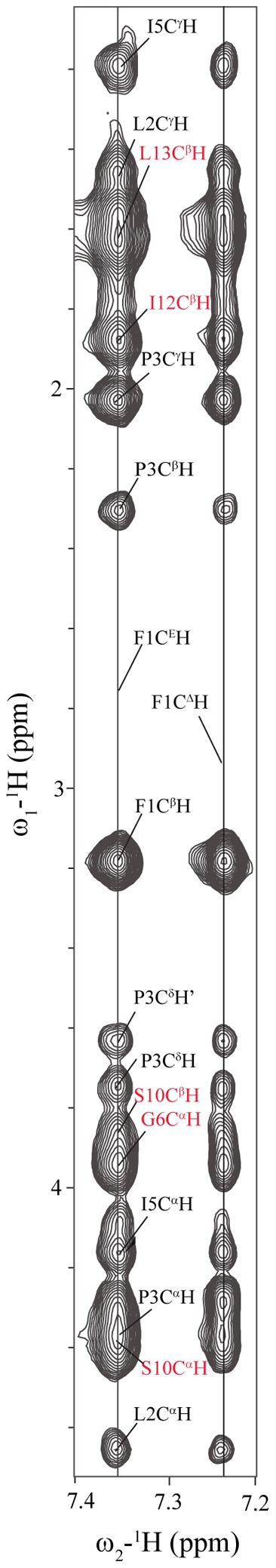
NOEs of residue F1 in LPS. A section of 2-D NOESY spectrum of TA, in aqueous solution containing LPS micelles, showing NOE connectivites from aromatic ring protons of residue F1 (along ω2 dimension) with the upfield shifted aliphatic proton resonances (along ω1 dimension). The long-range NOEs are indicated in red color.

**Figure 3:**
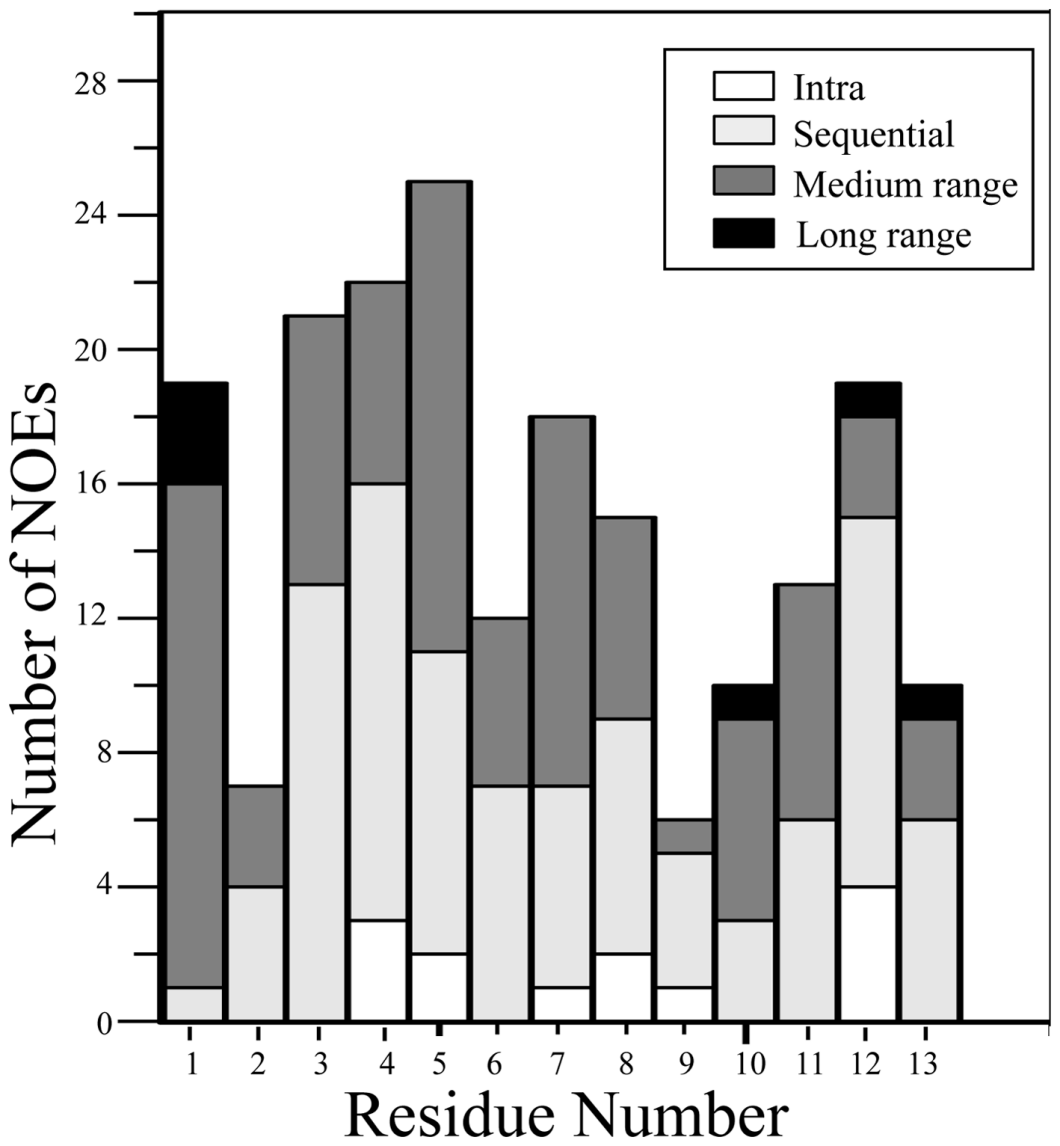
Summary of NOE contacts of TA in LPS micelles. Bar diagram summarizing type (intra, sequential, medium-range) and number of NOE contacts observed for each amino acid of TA while bound to LPS micelles.

The NOESY spectra of TA in solution showed only few NOE cross-peaks, indicating random conformations of the peptide (Figure S1). Atomic resolution structures of AMPs and LPS-binding peptides are determined in complex with LPS micelles by transferred nuclear Overhauser effect spectroscopy (tr-NOESY) [49], [50]. tr-NOESY experiments can be employed to obtain atomic resolution structures of peptide ligands while bound to the cognate high molecular weight targets [50]. LPS micelles are of high molecular weight and can be found at significantly low concentrations [51]. The critical micelle concentration of *E. coli* 0111:B4 LPS, used here, was estimated to be ∼ 1.3 to 1.6 µM [51]. Notably, tr-NOEs have been successfully used to determine 3-D structures of a number of potent AMPs in LPS micelles [23], [24], [25], [26], [27], [41]. One-dimensional proton NMR spectra of TA were acquired at various concentrations of LPS. Proton NMR spectra of TA showed broadening of almost all the resonances upon addition of LPS (Figure S2). The chemical shifts of the peptide did not exhibit any changes in the presence of LPS micelles (Figure S2). Two-dimensional tr-NOESY spectra of TA, in the presence of LPS micelles, revealed a large number of NOE cross-peaks (Figure 1). These observations, LPS induced line broadening and tr-NOEs, suggested a fast exchange of TA between the free and LPS-bound states. Analyses of 2-D tr-NOESY spectra of TA delineate intense sequential backbone NH/NH NOEs for residues L4-L13 (Figure 1A). A number of medium range NH/NH (i to i+2) and CαH/NOE NOEs (i to i+2, i+3 and i+4) are detected for several residues of TA (Figures 1A and 1B). These NOEs, sequential and medium range, correlating backbone resonances of TA in LPS micelles are diagnostic of the helical conformation. Further, the chemical shift of CδH_2_ proton resonances of residue P3 are non-degenerate and show NOEs with the NH resonance of residue L2 (Figure 1B). This observation indicates that the peptide bond between L2-P3 favors a *trans* conformation over a *cis* conformation. Interestingly, there are a number of NOE contacts involving the aromatic ring protons of residue F1with the upfield shifted backbone and sidechain proton resonances (Figure 2). In addition, the F1 ring resonances also exhibit NOEs with the backbone amide protons. Most strikingly, long-range NOEs are identified between aromatic ring protons of residue F1 with the CαH and CβHs of Ser10, CαH and CβH of Ile12 and amide proton of residue L13 (Figure 2). Figure 3 summarizes NOE contacts observed for TA in LPS micelles. Residues L2, P3 and I4 show as many as >20 NOEs (Figure 3). Notably, despite located at the very terminus, residue F1 shows a number of medium range and long range NOE interactions.

### Structure determination of TA in LPS micelles

**Figure 4:**
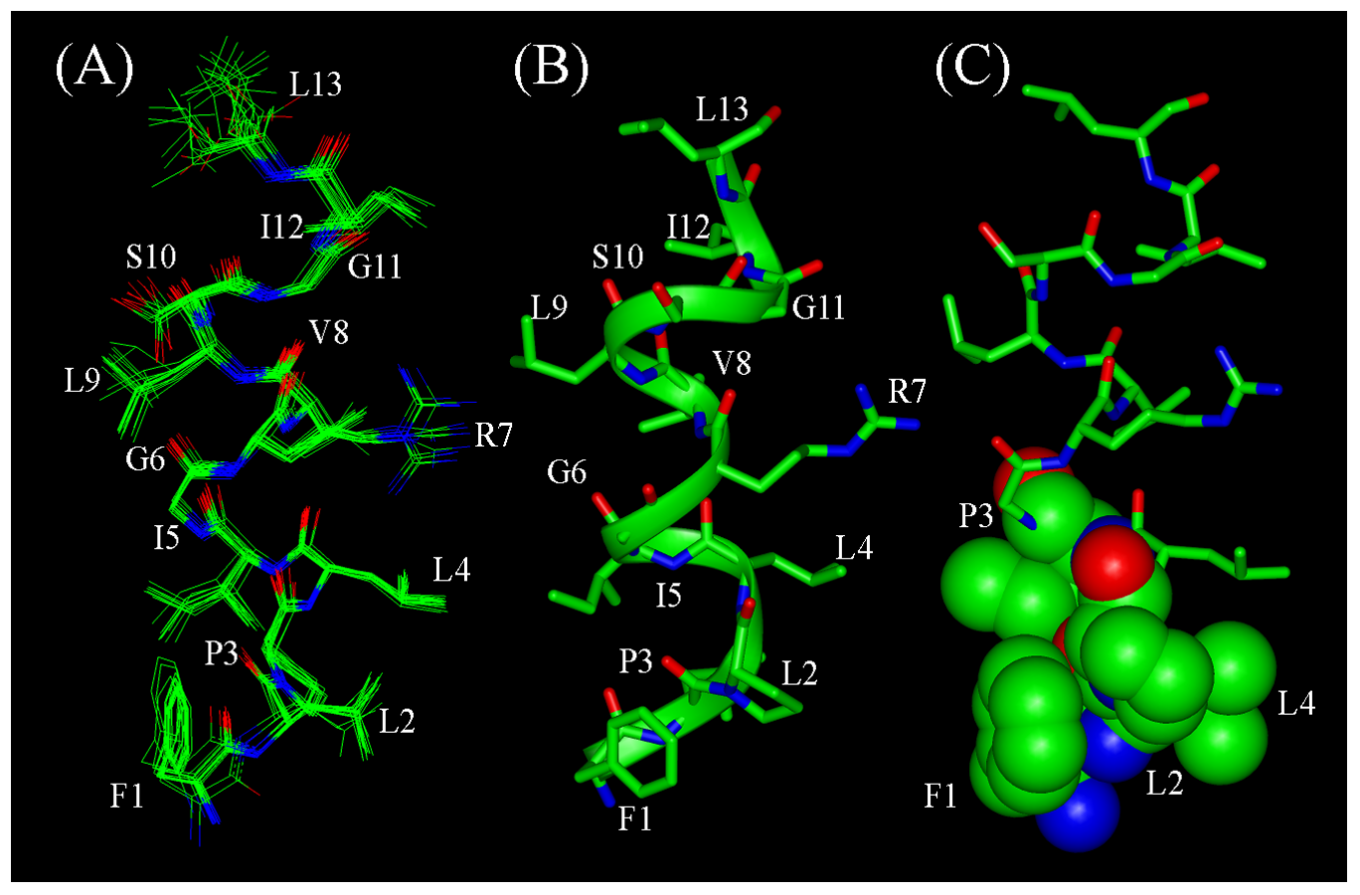
Three-dimensional structure of TA in LPS micelles. (panel A) Superposition of backbone atoms (N, Cα, C′) of twenty lowest energy conformers of TA for residues 1–13. (panel B) A representative conformation of LPS-bound TA showing sidechain orientation. (panel C) A space-fill representation of plausible packing interactions among the N-terminal residues, F1, L2, P3 and L4, of TA in LPS micelles. Figures were generated by INSIGHT II.

**Figure 5:**
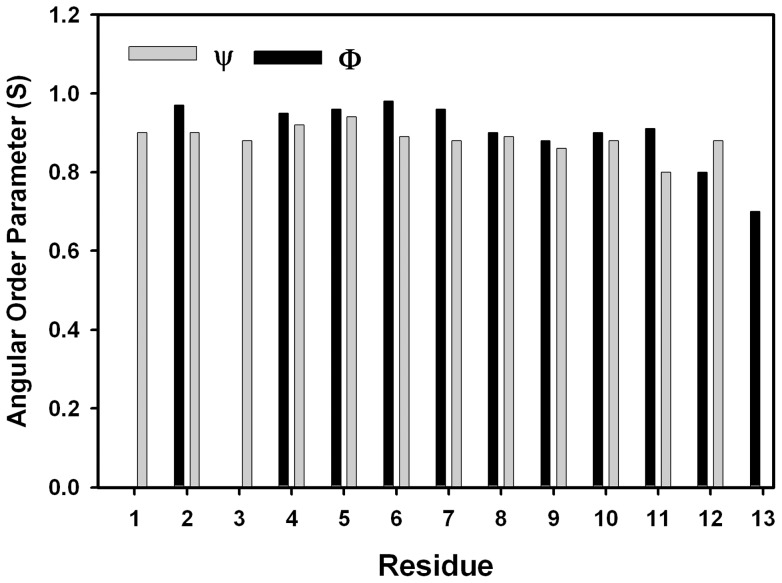
Angular order parameter (S) of TA structure in LPS. A bar diagram showing angular order parameter of backbone dihedral angles, Φ and Ψ, obtained from conformational ensemble of TA in LPS micelles.

**Figure 6:**
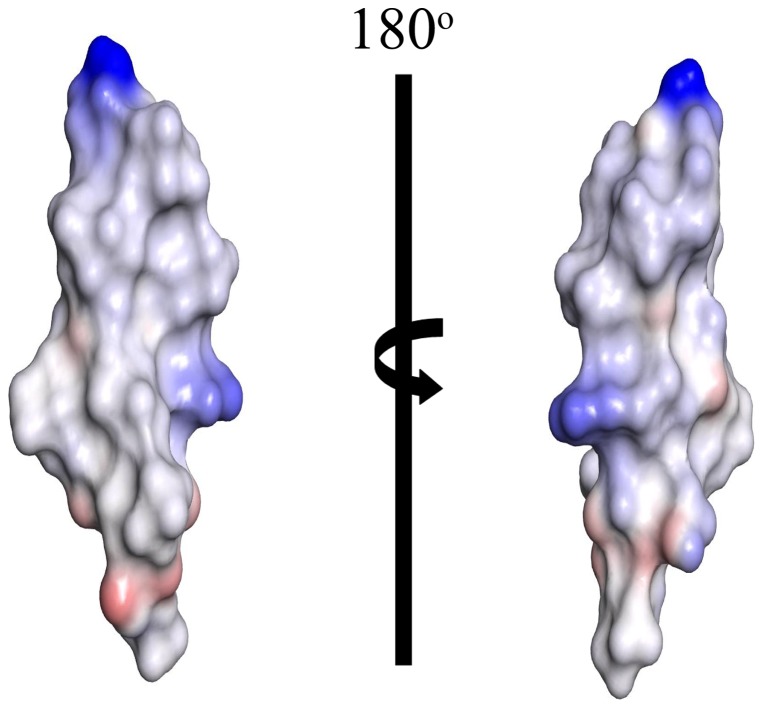
Electrostatic surface potential of TA in LPS micelles. Surface charge distribution of the helical structure of TA. Surfaces in red, blue and white represent, respectively, negatively charged, positively charged and neutral residues. The figure was generated by PyMOL.

LPS micelles bound structures of TA are determined based on the distance restraints obtained from the medium range and sequential NOEs (Table 1). The structures were further refined using backbone dihedral angle restraints for the non-glycyl residues (Table 1). Figure 4A shows superposition of twenty low energy structures of TA in LPS micelles. RMSD values of the backbone atoms and all heavy atoms are restricted to 0.32+0.20 Å and 0.75+0.30 Å, respectively. TA adopts helical conformation in LPS micelles for residues L4-I12 (Figure 4B). The residues L2 and P3 are in extended conformations (Figure 4B). Figure 5 shows the angular order parameter (S) of backbone dihedral angles (Φ, Ψ) of each residue of TA in LPS micelles. An S value of close to 1 for most of the residues of TA is indicative of a limited conformational sampling of the backbone in complex with LPS micelles. Strikingly, the 3-D structure of TA in LPS micelles reveals extensive mutual packing interactions among the sidechains of residues of F1, L2, P3, L4 and I5 (Figure 4C). The benzyl ring of F1 can be seen in a close contact with the pyrrolidine ring of P3 (Figure 4A and Figure 4B). The alkyl sidechain of residue L2 is in proximity with the sidechains of residues L4 and I5 (Figure 4A and Figure 4B). Such a juxtaposition of these sidechains appears to be sustaining a well defined extended nonpolar surface the N-terminus half of TA in LPS micelles (Figure 4C). There are diminished inter-sidechain packing interactions for residues G6-I12 of TA, since this segment contains residues G6, G11, R7 and S10. The sidechains of R7 and S10 are located at the opposite faces of the helical structure without any potential of forming polar interactions (Figure 4B). The nitrogen atom of NεH group of the guanidinium moiety of R7 is in close proximity, ∼3.4 Å, with one of the backbone alpha protons of residue G11. This may account for a possible hydrogen bonding between the sidechain of R7 and G11 of the TA helix in LPS micelles. More non-polar van der waals' packing can be realized among sidechains of residues V8, L9 and I12 for the TA helical structure. Electrostatic potential surface of the helical structure of TA in LPS micelles is largely defined by an extended non-polar surface with a positively charged region at the middle due to single cationic residue R7 (Figure 6).

**Table 1:** Summary of structural statistics of the twenty lowest energy structures of LPS-bound monomeric TA in aqueous solution.

**Distance restraints**	Intra-residue (|i–j| = 0)	18
	Sequential (|i–j| = 1)	43
	Medium range (2≤|i–j|≤4)	43
	Total	104
**Distance constraints violations**	Number of violation	4
	Average NOE violation (Å)	<0.24+0.2
	Maximum NOE violation (Å)	<0.50+0.12
**Deviation from mean structure**	Backbone atoms (N, Cα, C′) (Å)	0.32+0.20
	Heavy atoms (Å)	0.75+0.30
**Ramachandran plot analysis**	% residues in the most favorable region	90
	% residues additionally allowed region	10
	% residues in the generously allowed region	0
	% residues in the disallowed region	0

### Oligomerization of TA in LPS Micelles

**Figure 7:**
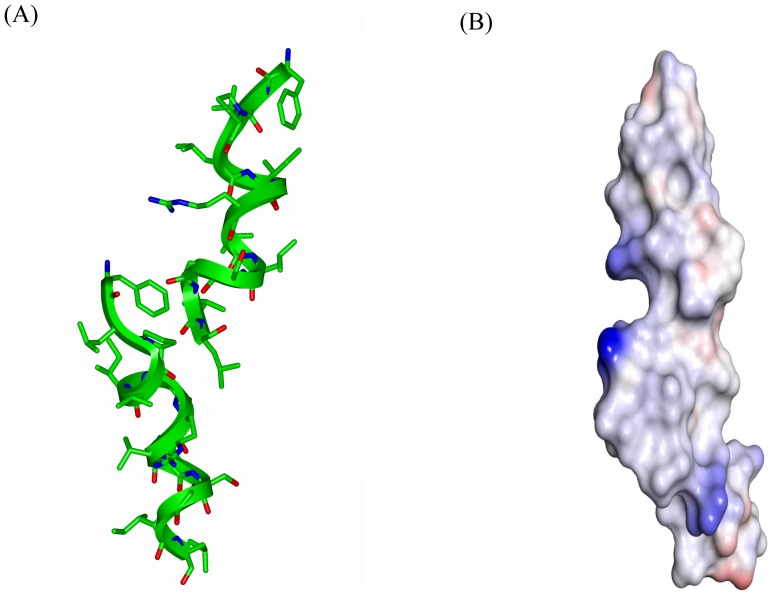
Mode of oligomerization of TA in LPS micelles. A model of the head-tail dimer of the helical structure of TA (panel A) and the electrostatic surface of the dimeric structure (panel B).

tr-NOESY spectra have revealed presence of a number of long-range NOEs involving residues at the N- and C-termini of TA in LPS micelles (Figure 3). The long-range NOEs are summarized in Table 2. These NOEs may putatively indicate formation of a closed symmetrical dimer of TA on LPS micelles. We have determined a dimeric anti-parallel helical structure of TA using the short and long-range NOEs derived distance constraints. However, the obtained dimeric structure appeared to be incompatible with the short inter-proton distances between two helical units. Firstly, the observed long-range NOE distance constraints could not be maintained in a single dimeric structure, since there were large distance constraint violation of 1 Å or higher. Secondly, there were a number of short inter-proton distances (<5 Å) e.g. CαH/CαH of G6/G11, CαH/CαH P3/G11, in the anti-parallel dimeric structures, however, corresponding NOE cross-peaks involving these protons were not detectable in tr-NOESY spectra. Thus, it is likely that TA may form an open helical dimer in LPS micelles employing residues from the N- and C-termini (Figure 7). Several of these head to tail dimeric units may potentially form helical oligomers in LPS lipid. It may also be worthwhile to consider that closed dimeric or oligomeric structures of TA would form an active AMP as observed for TL [41], beta defensin3 [52], cathelicidins [53], [54] and MSI-78 [55]. At this point, the exact size of the oligomers of TA formed in LPS is not clear. Although, such oligomers may be transient in nature and dependent on LPS structures. This proposition is in line with previous studies whereby TA was found to be forming oligomeric states in LPS and oligomeric states are disrupted by synergistic effect of TL [39], [40].

**Table 2:** Long-range NOEs of TA in LPS micelles.

Phe1 ring protons	Ser10 CαH
Phe1 ring protons	Ser10 CβHs
Phe1 ring protons	Ile12 CβH
Phe1 ring protons	Leu13 CβHs
Phe1 ring protons	Leu13 NH
Pro3 CβHs	Leu13 NH
Leu4 HN	Ile12 CβH
Pro3 CδHs	Ile12 CβH

### Saturation Transfer Difference (STD) NMR of TA in LPS Micelles

**Figure 8:**
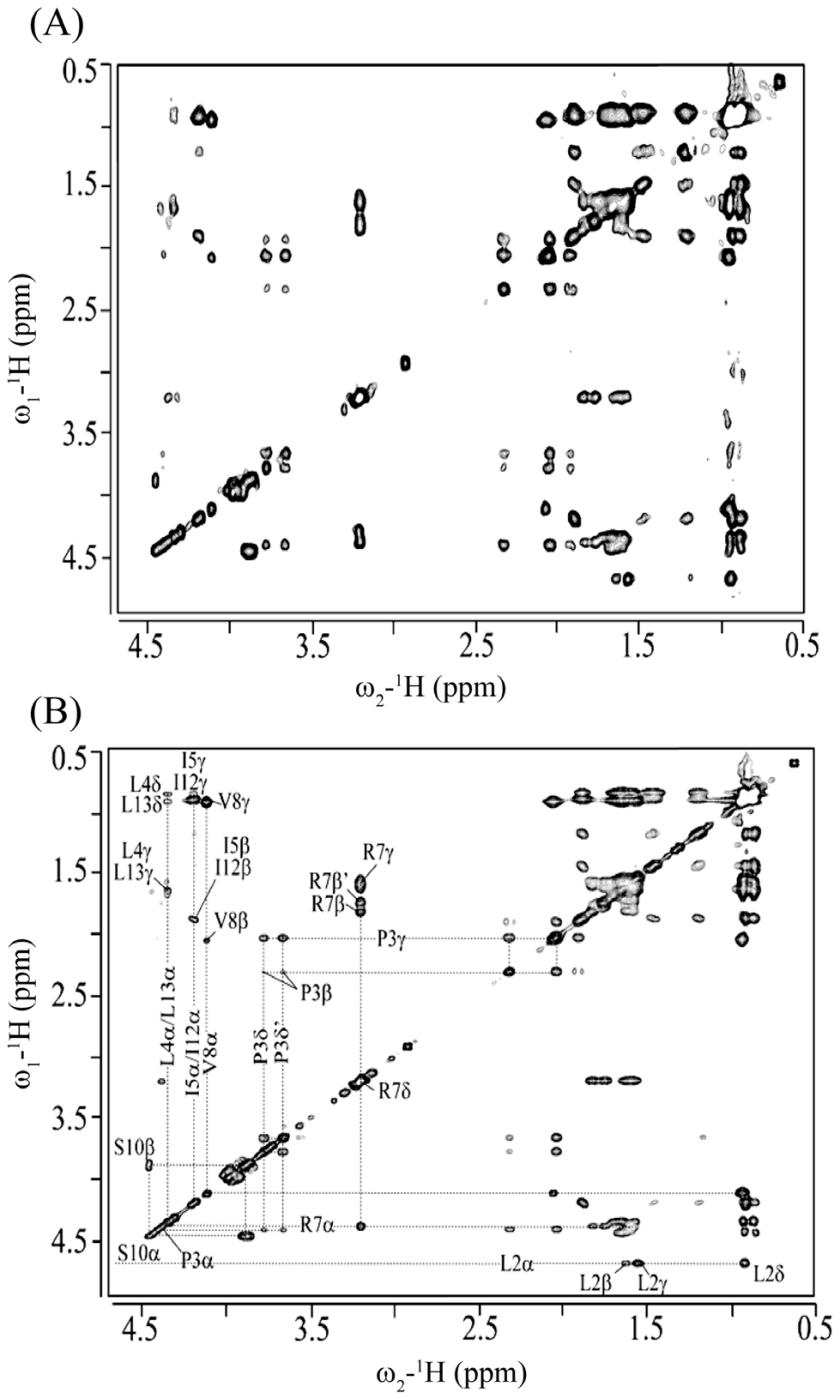
Localization of TA in LPS micelles by STD NMR. The off-resonance or reference TOCSY spectrum (panel A) and the STD-TOCSY spectrum (panel B) of TA in LPS showing correlations among aliphatic proton resonances. Through bond connectivites detected in STD-TOCSY spectra for amino acids residues of TA are shown by broken lines. STD-TOCSY spectra were acquired in D_2_O using a spin-lock MLEV17 sequence with a mixing time of 80 ms.

We have utilized STD-NMR to elucidate binding residues of TA with LPS. Figure 8 compares the off-resonance or the reference TOCSY spectrum (Figure 8A) with the STD-TOCSY spectrum (Figure 8B) of TA in LPS micelles. TOCSY correlations can be seen for almost all the residues including the CαH proton to the sidechain proton resonances and among the sidechain protons in the STD spectrum (Figure 8B). The benzyl ring protons of residue F1 also demonstrate intense STD TOCSY cross-peaks (Figure S3). These observations are indicative of intimate association of TA with LPS micelles. Similar STD spectra were also obtained for other potent AMPs including polymyxin B [56], pardaxin [27] and MSI-594 [23]. Although, unlike TA, these AMPs do not oligomerize in LPS micelles. The single cationic residue R7 of TA delineates STD TOCSY cross-peaks involving CδHs with CβHs and CγHs (Figure 8B). The CαH resonance of R7 also exhibits long-range TOCSY correlations with its CδHs, suggesting close proximity of R7 with the LPS micelles (Figure 8B). A strong cross-peak between CαH and CβH resonances of Ser 10 in the STD-TOCSY spectrum has elaborated polar interactions of TA with LPS micelles. Interestingly, residue P3 shows relatively strong STD-TOCSY cross-peaks from the CδH2 to CαH and CγH2, however, one of the CδH protons shows rather weak STD effect with CβbHs (Figure 8B). This observation may indicate a limited contact of P3 with LPS micelles. CαH and CβHs of residue F1did not yield cross-peaks in the STD spectra, implying probable lack of interactions of these protons with LPS lipid micelles. The TOCSY correlations from the CαH protons to sidechains can be seen for most of the aliphatic residues namely L2, L4, I5, V8, I12 and L13 of TA (Figure 8B), indicating their proximity with LPS lipids. Collectively, STD-NMR demonstrates that TA makes intimate interactions with LPS micelles. Strikingly, LPS-TA interactions not only involve the sidechains but also the backbone, CαH, atoms of the residues. This observation appears to be in contrast with LPS interactions of TB, whereby only limited STD effects were observed for the backbone atoms of the peptide [41].

## Discussion

Drug resistant bacteria represent a major concern in human health and infectious diseases; and the discovery of novel antimicrobials is highly demanding to combat the emergence of antibiotic resistant pathogens. Importantly, the short size of the temporin AMPs and the lack of toxicity (except for TL) towards human cells make them as attractive templates for the generation of new antibiotics [57], [58]. Among temporins, TL, TA and TB isoforms have been extensively studied for their structures, mechanisms of bactericidal activity and structure-activity relationships [38], [39], [46], [48]. Atomic resolution structures and interactions of temporins in/with LPS would highly help in understanding their limited activity against Gram-negative bacteria. We have recently investigated the structural organization and localization of TL and TB in LPS micelles [41]. TL assumes a dimeric helical structure whereas TB appears to be highly heterogeneous, probably resulting from self associations of the peptide in LPS [41]. As a result, atomic resolution structure of TB in LPS micelles could not be obtained. However, in the presence of TL, TB assumes monomeric helical structure in LPS micelles which is believed to assist TB to traverse the cell wall into the plamsa membrane. Note that such findings have provided the first structural insight into the molecular mechanism underlying the synergistic effect of TB+TL in the antimicrobial activity against Gram-negative bacteria.

NMR structures of TL and TA have been previously determined in SDS and DPC micelle solutions which are considered to be good models of anionic (bacterial) and zwitterionic (eukaryotic) membranes, respectively [59], [60]. TL and TA adopt monomeric helical conformations while bound to these micelles [59], [60], as also found for temporin SHs [61], [62]. Interestingly, our studies have demonstrated a clear difference in the structural organization of temporin A in LPS compared to detergent environments. Indeed, in both SDS and DPC micelles solution, TA adopts largely helical conformations except for residues F1-P3 [59]. A lower convergence was observed for the N-terminal segment where by the aromatic ring of F1 appeared to be excursing a larger conformational space [59]. By contrast, the 3-D structure of TA in LPS micelles reveals a well packed N-terminal region involving aromatic ring of F1 and sidechains of residues L2, P3 and I5 (Figure 4). The clustering of N-terminal residues of TA may be playing critical roles in inter-molecular association of the peptide in LPS lipids. The helical structures and the oligomeric states of TA are embedded in LPS lipid micelles. The STD effects from the sidechains of aromatic/aliphatic and polar residues of TA implicated probable involvement of polar head groups and hydrophobic regions of LPS binding with the peptide. In conclusion, we have determined 3-D structure, oligomerization and localization of TA in LPS micelles. The current study provides the first structural insights of a temporin peptide that can be considered ‘trapped’ in the LPS OM. The head to tail oligomerization of TA in the LPS layer could be responsible for the limited translocation of the peptide across the OM, and hence for its weak antibacterial activity. It is likely that systematic mutations of the residues involved in such oligomerizations may help in developing analogs of the non-toxic TA endowed with a potent activity also against Gram-negative bacterial strains.

## Materials and Methods

### Peptide and LPS

TA was commercially synthesized by GLBiochem^TM^ (Shanghai, China). *E. coli* 0111:B4 LPS, trichloroacetic acid extracted, was purchased from Sigma. The molecular mass of LPS was considered to be 10 KD [63].

### NMR spectroscopy

All NMR experiments were performed on a BRUKER DRX 600 spectrometer equipped with a cryo-probe and pulse field gradients. NMR data were collected at 298 K using DSS (2,2-dimethyl-2-silapentane-5-sulfonate sodium salt) as an internal standard (0.0 ppm). NMR spectra were processed with Topspin software (BRUKER) and analyzed using SPARKY (Goddard, T. D., and Kneller, D. G., University of California, San Francisco) running on a Linux workstation. Two-dimensional NOESY spectra (mixing time:150 ms) of free TA were acquired in an aqueous solution containing 10% D_2_O at pH 4.5 with peptide concentration of 0.5 mM. A series of 1-D ^1^H NMR spectra of TA were recorded with various concentrations of LPS, ranging from 2-10 µM, 2-D tr-NOESY experiments were performed, at 0.5 mM of peptide concentration, in the presence of 5 µM LPS, with four different mixing time: 50, 100, 150, and 200 ms. Spin systems of the peptide were assigned by 2-D TOCSY spectra (mixing time: 80 ms).

For Saturation transfer difference (STD) experiments, peptide TA (0.5 mM) and LPS samples, were prepared in D_2_O and pH was adjusted to 4.5. STD experiments were performed at 298 K in the presence of 5.4 µM LPS using standard STD pulse sequences [64] and WATERGATE 3-9-19 sequence for water suppression. STD experiments were carried out as described previously [27], [41]. On-resonance frequencies were fixed at −2.5 ppm. The off-resonance frequency for STD experiments was set at 40 ppm. 2-D STD-TOCSY spectra were recorded with 350 increments in t_1_ and 80 transients using a MLEV-17 spin lock field of 80 ms. The relaxation delay was fixed to 2.1 s. 40 selective Gaussian 270 pulses with duration of 50 ms were applied to achieve saturation transfer.

### Structure determination

An ensemble of monomeric helical structures of TA was determined using the CYANA program [65]. 2-D tr-NOESY spectra obtained at 150 ms mixing time were utilized to generate the distance constraints. Based on the intensity of NOE cross-peaks upper bound distance restraints were categorized to 2.5 Å, 3.5 Å and 5 Å. The lower distance was fixed to 2.0 Å. For the structure calculation of the dimeric TA, all of the long range NOE contacts were assigned to an upper bound distance of 5 Å. Out of 100 structures, 20 structures with the lowest target function were selected to represent the ensemble. Angular order parameter (S) values for backbone dihedral angles (Φ, Ψ) were calculated following previous work [26]. Structures were visualized using MOLMOL, pyMOL, and Insight II software. The quality of structures was determined using Procheck [66] and Protein Structure Validation Suite [67]. NMR chemical shifts and structural coordinates of TA have been deposited to BMRB and Protein Data Bank with accession number, 19334 and 2maa, respectively.

## Supporting Information

Figure S1 **Conformations of TA in free solution.** A section of 2-D NOESY spectrum of TA in aqueous solution, pH 4.5, 298 K.
(TIF)

Figure S2 **Interactions of TA with LPS micelles.** Low field region of 1-D NMR spectra of TA in free solution (lower panel), in 3 µM LPS (middle panel) and in 5 µM LPS (top panel) in aqueous solution, pH 4.5, 298 K. The amide proton resonances (7.9–8.4) and aromatic ring proton resonances of F1 are marked. Other resonances in 7.4–6.9 ppm are arising from sidechain of Arg7 and amide groups of the C-terminus.
(TIF)

Figure S3 **Interactions of TA with LPS micelles by STD.** A section of 2-D STD-TOCSY spectrum of TA in LPS micelles showing STD effect for ring protons of the F1 residue.
(TIF)
