# Supplementary figures and images for: NMR Structure of Temporin-1 Ta in Lipopolysaccharide Micelles: Mechanistic Insight into Inactivation by Outer Membrane
Source: PLoS One. 2013 Sep 9;8(9):e72718. doi: 10.1371/journal.pone.0072718 (PMC3767682; doi:10.1371/journal.pone.0072718)

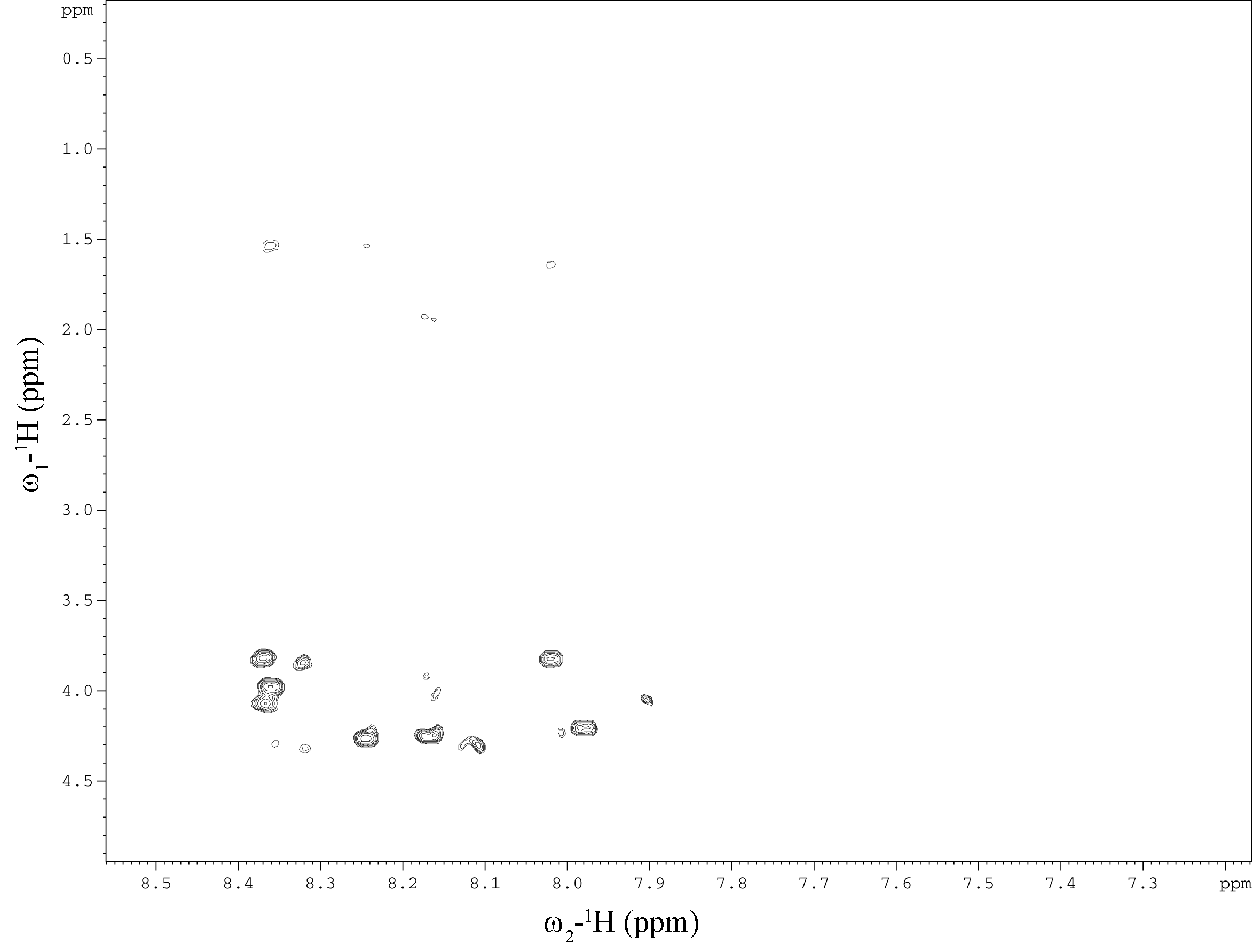

Supplement: Figure S1 — Conformations of TA in free solution. A section of 2-D NOESY spectrum of TA in aqueous solution, pH 4.5, 298 K. (TIF) [file pone.0072718.s001.tif]

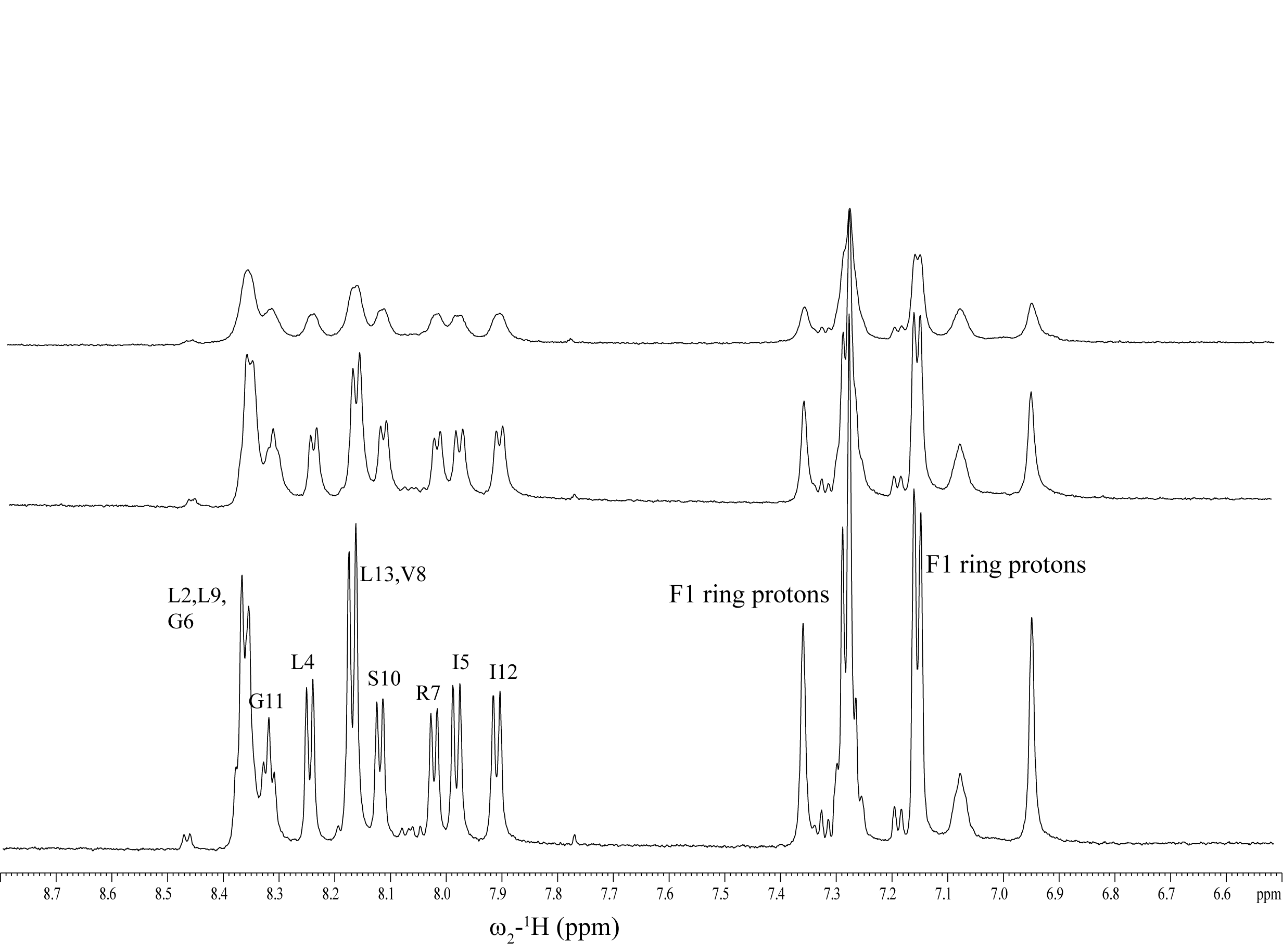

Supplement: Figure S2 — Interactions of TA with LPS micelles. Low field region of 1-D NMR spectra of TA in free solution (lower panel), in 3 µM LPS (middle panel) and in 5 µM LPS (top panel) in aqueous solution, pH 4.5, 298 K. The amide proton resonances (7.9–8.4) and aromatic ring proton resonances of F1 are marked. Other resonances in 7.4–6.9 ppm are arising from sidechain of Arg7 and amide groups of the C-terminus. (TIF) [file pone.0072718.s002.tif]

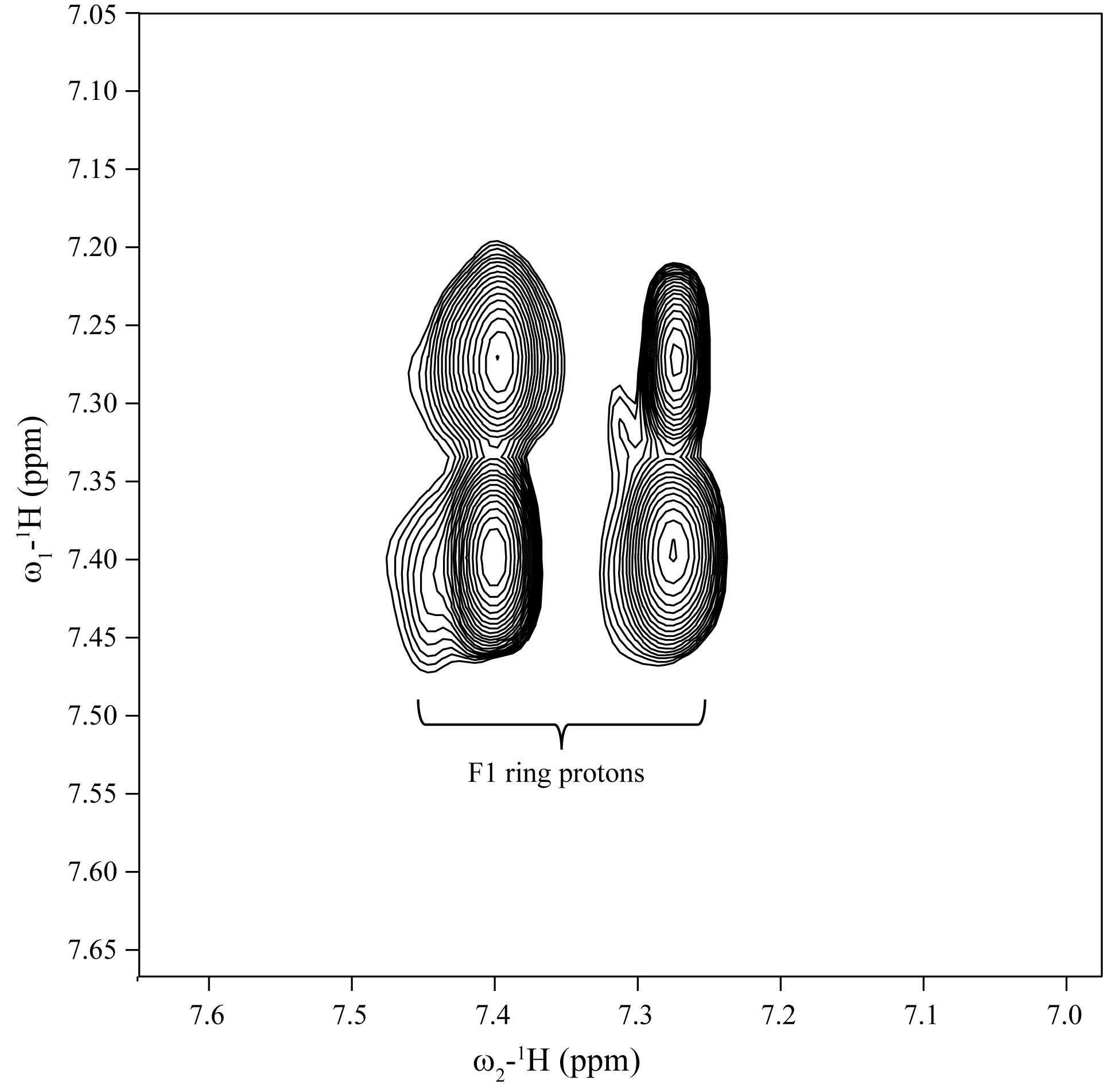

Supplement: Figure S3 — Interactions of TA with LPS micelles by STD. A section of 2-D STD-TOCSY spectrum of TA in LPS micelles showing STD effect for ring protons of the F1 residue. (TIF) [file pone.0072718.s003.tif]
